# Dementia Home Care in Urban and Rural India: A qualitative exploration

**DOI:** 10.1002/alz70858_102776

**Published:** 2025-12-25

**Authors:** Soja Joseph, Faheem Arshad, Priya Treesa Thomas, Suvarna Alladi

**Affiliations:** ^1^ National Institute of Mental Health and Neurosciences [NIMHANS], Bengaluru, Karnataka, India; ^2^ National Institute of Mental Health and Neurosciences, Bengaluru, Karnataka, India

## Abstract

**Background:**

Dementia home care in urban India faces significant challenges, including social stigma, inadequate resources, lack of knowledge about the resources, lack of help seeking behaviour and caregiver burden. Despite these hurdles, home care is a major setting where interventions including personalized support, improved quality of life and dignity can be provided, enabling family members to balance caregiving responsibilities with work and obligations. This study aims to understand the beliefs, stigma and perceptions regarding home care services among the rural and urban population receiving home care.

**Method:**

A mixed method, content analysis study was conducted with the people living with dementia cared for in their own homes by family members, who were referred to the neuropalliative services and received home‐care. The participants were recruited for the study if they had an established diagnosis of dementia; had a CDR score of 1 Based on an intake need assessment (sociodemographic proforma, caregiver burden and palliative care needs), the multidisciplinary team visited the participants at home. Detailed case notes were maintained, and the team members maintained reflexive field notes after each visit. The notes were thematically analysed and triangulated.

**Result:**

In this study twenty people living with dementia, 10 from Urban and 10 from Rural areas who were part of the home care neuropalliative services were included. The mean age of the study population is 62.2 (11.90); with more female preponderance. The primary caregiver mean age is 45.65 (SD‐ 13.5) The caregivers reported moderate to severe caregiver burden scale is 47.75 (SD‐17.09) and post assessment zarit caregiver burden mean score is 37.95 (SD‐15.77). Integrated Palliative outcome score‐ dementia was 22.8 (SD‐8.60). This study revealed significant gaps in dementia care knowledge among caregivers. While participants demonstrated awareness of hospital‐based treatment, they lacked understanding of home‐based care strategies, caregiving responsibilities and available resources and multidisciplinary approach in dementia care. Significant differences in knowledge, attitude, caregiving practices and help seeking behaviour was observed among the rural and urban participants.

**Conclusion:**

Dementia home care support is vital for urban Indian communities. Tailored interventions enhance caregiver knowledge and confidence. A multidisciplinary approach targets increasing awareness and accessibility of resources, and mitigates psychosocial burden.

